# Immune Regulation during Chronic Visceral Leishmaniasis

**DOI:** 10.1371/journal.pntd.0002914

**Published:** 2014-07-10

**Authors:** Rebecca J. Faleiro, Rajiv Kumar, Louise M. Hafner, Christian R. Engwerda

**Affiliations:** 1 QIMR Berghofer, Brisbane, Australia; 2 Queensland University of Technology, Brisbane, Australia; University of Texas Medical Branch, United States of America

## Abstract

Visceral leishmaniasis is a chronic parasitic disease associated with severe immune dysfunction. Treatment options are limited to relatively toxic drugs, and there is no vaccine for humans available. Hence, there is an urgent need to better understand immune responses following infection with *Leishmania* species by studying animal models of disease and clinical samples from patients. Here, we review recent discoveries in these areas and highlight shortcomings in our knowledge that need to be addressed if better treatment options are to be developed and effective vaccines designed.

## Introduction

Leishmaniasis is a disease caused by protozoan parasites belonging to the genus *Leishmania*. It affects people and animals in all parts of the world and can be broadly divided into visceral and cutaneous forms. Pathogenesis and immunity associated with cutaneous leishmaniasis (CL) have recently been reviewed [1] and will not be discussed here. Instead, we will focus on immune regulation during chronic visceral leishmaniasis (VL).

VL is a potentially fatal human disease with an estimated incidence of at least 0.2 to 0.4 million cases worldwide, causing 20,000–40,0000 deaths each year [2]. Around 90% of VL cases occur in six countries: India, Ethiopia, Bangladesh, Sudan, South Sudan, and Brazil [2]. It should be noted that these numbers are likely to be gross underestimates due to poor reporting and misdiagnosis [3]. In addition, data from epidemiological studies indicate that only 1 in 5–10 infected individuals develop clinical VL [4]–[6], suggesting the number of infected individuals, and hence potential parasite reservoirs, is far greater.

VL is caused by the obligate intracellular protozoan parasites *Leishmania donovani* in humans and *L. infantum (chagasi)* in both humans and dogs [7]. The parasite is transmitted by female *Phlebotomine* sandflies as a flagellated, metacyclic promastigote, which is phagocytised by host macrophages and then differentiates into the nonflagellated, replicative amastigote [8]. Amastigote numbers increase via binary fission and ultimately cause the bursting of the host cell, allowing the released parasites to infect other phagocytic cells [9]. The organs commonly affected during VL are the bone marrow, liver, and spleen [8]. Clinical symptoms include hepatosplenomegaly, which is characterized by an enlarged abdomen with palpable spleen and liver. Other symptoms include long-term, low-grade fever, muscle wasting, anaemia, leukopenia, polyclonal hypergammaglobulinemia, and weight loss [10], [11]. Mucosal haemorrhage, and ultimately sepsis, may also occur as a result of loss of prothrombin and thrombocytes. VL is almost always fatal if left untreated. Hyperpigmentation of warmer regions of the body is commonly observed in Indian patients, hence the derivation of the name *kala-azar*, meaning black fever in Hindi [12]. The diagnosis of VL is confirmed by microscopic demonstration of amastigotes in spleen or bone marrow biopsies. Serological tests, such as rK39 dipsticks, are also used for diagnosis but with the limitation that they cannot differentiate between past and present infection. Polymerase chain reaction (PCR) is another potential diagnostic option [13] but has not been established for use in field settings where VL is endemic.

Many VL patients become severely immunocompromised and can succumb to secondary infections [14]. This was thought to be associated with their inability to generate cell-mediated immune (CMI) responses against previously encountered antigens, reflecting an accumulation of dysfunctional T cells [5]. However, as discussed below, recent studies suggest that some findings relating to T cell nonresponsiveness might be attributed to the particular experimental approaches employed. Regardless, the functional capacity of antigen-presenting cells (APCs) is compromised in these patients [8]. Furthermore, many VL patients also produce high levels of the suppressive cytokine interleukin-10 (IL-10), which can inhibit the activity of antiparasitic proinflammatory cytokines such as interferon gamma (IFNγ) and tumour necrosis factor (TNF) [15]. At present, there is no effective vaccine to prevent or treat VL in humans [16]. In addition, drug treatment is undermined by toxicity in patients and increasing frequencies of drug-resistant parasites [17].

## Methods

References for this article were identified through PubMed searches for articles published from 1982 to 2013 using the terms “*Leishmania*,” “*donovani*,” “*infantum*,” “human,” “immune regulation,” “visceral leishmaniasis,” “T cell,” “dendritic cell,” “monocyte,” “neutrophil,” “cytokine,” “chemokine,” and “vaccine.” Relevant books and articles published between 1965 and 2013 were selected through searches in the authors' personal files.

### Past and present treatments and prevention

The most common VL treatment for the last 60 years has been antimonial chemotherapy [18]. Pentavalent antimonials, such as sodium stibogluconate, pentostam, meglumine antimonite, and glucantime, have been the mainstay of antimonial therapy [19]. However, there is now considerable parasite resistance against these drugs, especially in northeastern India and surrounding areas [17]. Therefore, although these drugs are still employed to treat VL in Africa, drugs such as Amphotericin B, Ambisome (lipid formulation of Amphotericin B), Miltefosine, and aminosidine (paromomycin) have been developed as alternative treatments against VL in areas of antimonial drug resistance [17]. However, these drugs are still far from ideal because of cost, toxicity, the development of parasite drug resistance after prolonged use, and the duration of treatment times [20]. However, some progress has been made recently in addressing this final issue, as a single dose of Ambisome was found to be sufficient to successfully treat VL and has now been recommended as a choice of treatment in India [21], [22].

The development of a vaccine to prevent leishmaniasis has been a long-term goal for many researchers. In theory, a vaccine to prevent leishmaniasis should be possible, as indicated by past programs of leishmanisation. This process involves the deliberate infection of people with CL-causing parasite species on unexposed areas of the body to establish an infection that is controlled in most individuals, resulting in long-term protection [23]. This technique was practised for centuries throughout the Middle East and in parts of Asia, and large-scale trials were carried out in the former Soviet Union and Israel with some success [24], [25] as long as the parasites used were viable and infective [26]. However, despite the solid immunity that develops in most individuals, this approach has largely been abandoned because some individuals develop complications, such as large skin lesions, exacerbation of skin diseases, and poor response to other vaccines [27], [28]. To the best of our knowledge, leishmanisation has not been tested to prevent VL in humans, but a recent report showed that infection of BALB/c mice with a naturally attenuated *L. donovani* strain isolated from skin lesions of a CL patient in Sri Lanka conferred protection against a visceralising strain of *L. donovani* [29].

The vaccines currently being developed against VL can be divided into three groups: first, there are vaccines which involve vaccination with live-attenuated or killed parasites; second, there are vaccines which involve genetically modified parasites, subunit vaccines, or recombinant parasite proteins produced by virus or bacteria; and third, there are vaccines which consist of plasmid DNA and viral-based vaccines encoding immunogenic *Leishmania* proteins [16], [30], [31]. One of the major hurdles for developing vaccines to either prevent or treat VL has been a limited understanding of the precise immune mechanisms required for controlling parasite growth without causing disease. Because of the intrusive techniques required to analyse tissue in VL patients, our current understanding of the host immune response during VL largely derives from studies performed in *L. donovani–*infected, genetically susceptible mice.

### Experimental VL

Lifelong, chronic infection can be established experimentally by intravenous injection of *L. donovani* amastigotes into genetically susceptible mice [32]. Resistance and susceptibility to *L. donovani* infection in mice is controlled by the *Slc11a1* gene (formerly Nramp1—“natural resistance associated macrophage protein 1”) present in both mice and humans [33]. This gene encodes an iron and manganese transporter involved in the activation of macrophage antimicrobial mechanisms. Genetically resistant mice have a functional *Slc11a1* gene, while susceptible mice have a naturally occurring Glysine → Aspartic–acid amino acid mutation, resulting in a nonfunctional *Slc11a1* gene [8]. BALB/c and C57BL/6 mice are genetically susceptible to *L. donovani* infection and are commonly used for experimental studies. *Leishmania* infection in these mice is nonfatal, and the immune-related tissue pathology observed shows some similarity to the spectrum of clinical symptoms reported in VL patients [34].

#### Organ-specific immune responses

Genetically susceptible mice infected with *L. donovani* develop distinct, organ-specific immune responses as disease progresses [35]. The liver is the site of an acute and resolving infection, whereas a chronic infection becomes established in the spleen and the bone marrow (BM) [34], [36]. In the liver, *L. donovani* amastigotes multiply rapidly during the first 4 weeks of infection but are controlled by the 8th week of infection. In contrast, in the spleen and the BM, parasite numbers increase slowly over the first 4 weeks, and a persistent infection becomes established. This tissue-specific pattern of parasite growth appears to be common for all visceralising species and strains of *Leishmania* in genetically susceptible mice [37].

#### The establishment of immunity in the liver

**Figure 1:**
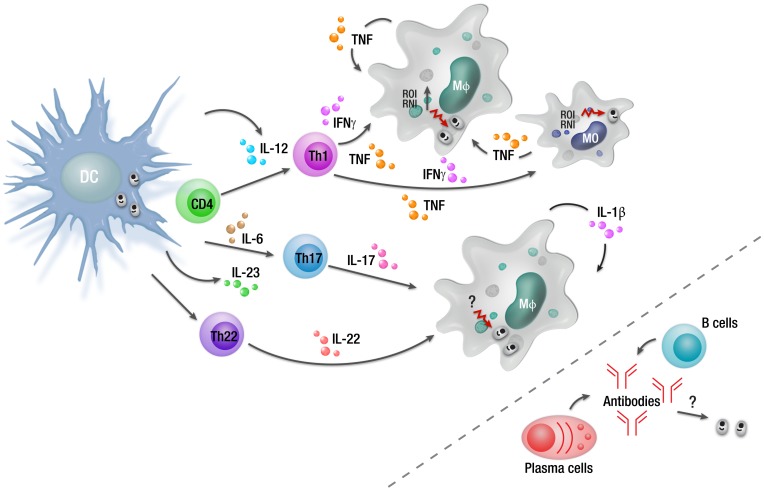
Overview of cellular responses during an asymptomatic *L. donovani* infection. Infected macrophages can produce TNF and IL-1β in response to *L. donovani* infection as part of the innate immune response. However, DC IL-12 production in response to *L. donovani* infection is required to drive the differentiation of antigen-specific CD4^+^ T cells into IFNγ- and TNF-producing Th1 cells. These cells can activate infected macrophages and monocytes to produce ROI and RNI that kill intracellular parasites. There are also reports in humans that Th17 and Th22 cells develop in asymptomatic, infected individuals, possibly driven by IL-23 and IL-6. However, the antiparasitic mechanism mediated by these CD4^+^ T cell subsets following *L. donovani* infection remains unknown. Although parasite-specific antibodies are readily detected in asymptomatic individuals, their role, if any, in control of infection and protection against reinfection is unknown. Abbreviations: MO, monocyte; Mφ, macrophage.

In the liver during experimental VL in genetically susceptible mice, parasitic burdens peak between weeks 2–4 of infection, and then parasite growth is controlled by weeks 6–8 postinfection, although sterile immunity is not achieved [7]. Control of hepatic infection depends on the formation of inflammatory granulomas [38]. Kupffer cells (KCs), the resident tissue macrophages in the liver, are the primary cells infected by *L. donovani* amastigotes [39]. Early chemokine and cytokine production by KCs is thought to recruit monocytes and neutrophils to the site of infection in the first few days after infection that further amplify chemokine production [40], [41]. Depletion of neutrophils early during *L. donovani* infection indicates that they play an important role in controlling parasite growth [42]. However, there is also strong evidence from models of CL that these cells may help establish infections by acting as a safe haven for parasites before being taken up by monocytes [43]. An important antiparasitic role for monocytes for early control of *L. donovani* infection has been established [40], [44], although this may be more complicated than first thought, given the plasticity of these cells and their ability to differentiate into potent APCs or regulatory cells [45], [46]. The recruitment of neutrophils and monocytes into the liver is followed by the recruitment of T cells, which are critical for efficient granuloma formation around infected KCs and control of parasite growth [47]. In particular, CD4^+^ T cells that have been activated by dendritic cells (DCs) producing interleukin 12 (IL-12) are critical to these processes via the production of proinflammatory cytokines, including IFNγ, TNF, and lymphotoxin alpha (LTα) [38], [48], [49]. These cytokines can further amplify cellular recruitment to infected KCs, as well as activate antimicrobial mechanisms in these cells [8]. These microbicidal mechanisms include the generation of reactive oxygen intermediates (ROI) and reactive nitrogen intermediates (RNI) that are both capable of killing parasites in infected macrophages, although only the latter is critically important for the resolution of the disease (Figure 1) [50]. Recent studies have identified three C-type lectin receptors as important pattern recognition receptors for *L. infantum* [51]. Dectin-1 and mannose receptor were found to play important roles in generating antiparasitic responses, in particular for ROI production. In contrast, specific intercellular adhesion molecule-3-grabbing non-integrin receptor 3 (SIGNR3; a homologue of human dendritic cell-specific intercellular adhesion molecule-3-grabbing non-integrin [DC-SIGN]), appeared to promote parasite persistence by inhibiting IL-1β production. Another study recently showed that early inflammasome-derived IL-1β was critical for the induction of RNI by *L. infantum*-infected macrophages [52], thus identifying critical early events in parasite recognition and control by the host.

After 4 weeks of *L. donovani* infection, well-organised and functional mature granulomas are observed in the liver, associated with the control of parasite growth and a decline in parasite burden [49]. Parasite numbers decline until 6–8 weeks postinfection, after which a relatively low-level persistent infection that is contained within granulomas by CD4^+^ T cells becomes established [53], [54]. Following reinfection, parasite growth is controlled within 1–2 weeks, with parasite burden only reaching a fraction of the primary infection, indicating the development of productive immunological memory that may include a CD8^+^ T cell component [39].

The proinflammatory environment that develops in order to control parasite growth in the liver of mice and in asymptomatic individuals infected with *L. donovani* (Figure 1) has the potential to cause tissue damage, as can be the case during chronic infection (see below). However, this does not occur, thereby suggesting the efficient generation of immune regulatory networks to protect tissues. Whether these networks can be distinguished from those that become established during chronic infection and contribute to parasite persistence and disease remains unknown but will be an important area of future research.

#### The development of a chronic infection in the spleen and bone marrow

*L. donovani* also infects tissue macrophages found in the spleen and BM [34], [55]. These initially include the marginal zone macrophages (MZMs) and marginal metallophilic macrophages (MMMs) in the spleen [56] and stromal macrophages in the BM [55]. In the spleen, subsequent infection of red pulp (RP) macrophages by parasites also occurs [57]. Despite a small drop in parasite burden in the spleen 24 hours after infection, parasites numbers increase and then stabilise over the following 1–2 months, leading to a chronic infection [56]. A similar pattern of parasite growth also occurs in the BM [58]. Chronic infection in the spleen leads to splenomegaly and results in structural alterations in the macroarchitecture of the spleen tissue [59], which are thought to contribute to immune suppression in this organ during VL [34], [59].

**Figure 2:**
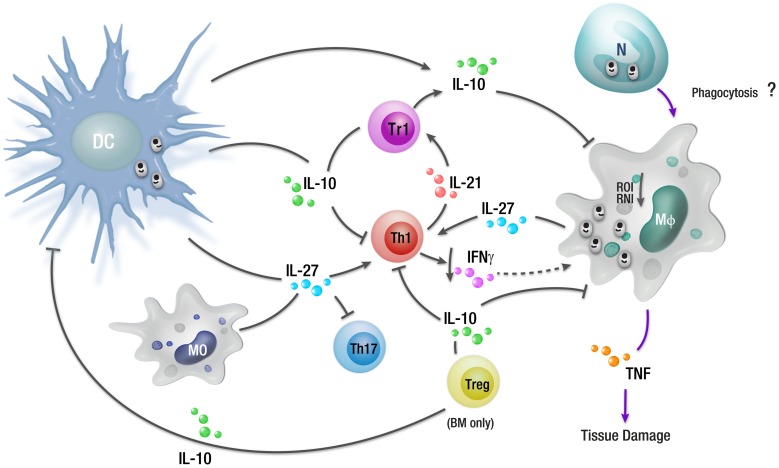
Overview of cellular responses during a chronic *L. donovani* infection. During an established *L. donovani* infection, a subset of regulatory DCs in the spleen can produce IL-10 that promotes the expansion of IL-10-producing regulatory T cells (Tr1), as well as inhibiting antimicrobial mechanisms in macrophages and other phagocytic cells (including suppression of ROI and RNI generation). IL-27 produced by regulatory DCs and macrophages, along with T cell–derived IL-21, can drive the differentiation of Th1 cells into Tr1 cells, as well as inhibit Th17 development. IL-10 produced by Tr1 cells can suppress antigen presentation, contributing to T cell dysfunction, as well as down-regulate CD4^+^ T cell IFNγ production. There has been a report that IL-10 can also be produced by Treg cells in the BM of VL patients. Although uptake of infected neutrophils undergoing apoptosis by macrophages contributes to the establishment of *L. major* infection in mice, no such mechanism has yet been described during *L. donovani* infection. Abbreviations: N, neutrophil.

The marginal zone (MZ) region of the spleen plays an important role in directing cellular traffic and is located between the macrophage beds of the RP and the T and B cell zones contained within the white pulp. MZMs and MMMs, sinus-lining reticular cells, B cells, and DCs, as well as blood migrating cell populations, make up the MZ region [60]. During a chronic *L. donovani* infection, widespread remodelling of the MZ region takes place, including the loss of MZMs and some displacement of MMM populations [59]. The loss of MZMs during VL is associated with disrupted lymphocyte migration into the white pulp of the spleen [59]. Within the white pulp region, remodelling causes disruption to both the gp38^+^ fibroblastic reticular cell (FRC) network, which guides T cell and DC migration to the T cell zone [61], and the follicular DC (FDC) network in the B cell follicles[34]. Work carried out by Dalton et al. showed that, by using a receptor tyrosine kinase inhibitor (RTKI) sunitinib maleate (Sm), vascular remodelling and splenomegaly associated with VL can be blocked and the effects can be reversed [62]. However, use of Sm alone did not cause a reduction in parasite burden in the spleen, but when used in combination with conventional antimonial drugs, enhanced leishmanicidal activity was observed [62]. *L. donovani* infection also appears to promote development of regulatory DCs in the spleen. Examination of DC populations in the infected spleen showed an increase in CD11c^l^°CD45RB^+^ DCs, compared to the CD11c^hi^ DC population [63]. DCs with the CD11c^l^°CD45RB^+^ phenotype secrete IL-10 when stimulated with lipopolysaccharide (LPS) and skew T cell development to IL-10-producing regulatory T cell (Tr1, producing both IFNγ and IL-10) responses (Figure 2) [64]. The development of these regulatory DCs was mediated by stromal cells in both naive and infected spleens [63]. CD11c^l^°CD45RB^+^ DCs show features of immature DCs, indicated by low expression of co-stimulatory molecules and intracellular major histocompatibility complex (MHC) class II [63]. These DCs were capable of inhibiting mixed lymphocyte reactions (MLRs) driven by conventional DCs, and this effect could be reversed by the presence of an anti-IL-10 receptor monoclonal antibody (mAb) [63]. Comparative analysis of regulatory DCs (CD11c^l^°CD45RB^+^) generated in the presence of naive spleen stromal cells and *L. donovani*-infected spleen stromal cells showed that the latter had elevated regulatory capacity which could overcome the effects of anti-IL-10 receptor mAb. In addition to inhibiting MLRs, these DCs also had elevated levels of IL-10 mRNA accumulation compared to CD11c^l^°CD45RB^+^ DCs generated in the presence of naive spleen stromal cells [63].

The TNF family of cytokines and their associated signalling molecules play an important role in the development of the splenic MZ region [65]–[69]. TNF is expressed throughout the spleen during established *L. donovani* infection and plays an important role in tissue remodelling and, in particular, in the breakdown in tissue microarchitecture. *L. donovani–*infected mice receiving TNF blockade, as well as TNF-deficient mice infected with *L. donovani*, had a reduced loss of MZM, compared with control animals, and although some structural changes were found in the spleens of these animals, they were far fewer compared to those found in control-infected mice [59]. One of the consequences of this overt TNF production and the subsequent impact on the MZ region is thought to be that DCs and naive T cells fail to migrate to the periarteriolar lymphoid sheath (PALS) of the spleen, resulting in reduced priming of naive T cells [7].

We and others have previously shown that mice deficient in IL-10 fail to establish a substantial *L. donovani* infection and that blockade of IL-10 signalling during an established *L. donovani* infection dramatically enhances antiparasitic immunity [8], [70], [71]. There is strong evidence that IL-10 plays a key role in regulating the expression of the programmed death (PD)-1 ligands (PD-L1 and PD-L2) on APCs [72], and there has been a report that the splenic environment during chronic VL is associated with the increased expression of PD-L1 on DCs [73]. Furthermore, following ligation of PD-L1 to its receptor PD-1 found on T cells, there is diminished T cell proliferation and cytokine production [74]. Blocking PD-L1 ligation during *L. donovani* infection results in increased survival of CD8^+^ T cells and also partially restores the functional capacity of these cells [7]. The partial restoration of CD8^+^ T cell functionality indicates that there may be several other important immune regulators that also suppress cytokine production by these cells.

IL-27 has been shown to play a major role in the induction of IL-10-producing T cells [75]. A study in mice revealed that IL-27 drives the expansion and differentiation of IL-10-producing Tr1 cells, promoting c-maf-mediated IL-21 production, which acts as an autocrine growth factor for the expansion and/or maintenance of IL-27-induced Tr1 cells (Figure 2) [76]. IL-27 belongs to the IL-12 cytokine family, and previously, IL-27Rα-deficient mice infected with *Toxoplasma gondii* were found to develop a normal T helper (Th) 1 response but then died when this response became severely dysregulated [77]. IL-27 has been reported to play critical roles in experimental *Leishmania* infection. IL-27Rα-deficient mice infected with *L. donovani* developed enhanced Th1 responses, but severe liver pathology was also observed in these mice [78]. In nonhealing *L. major* infection, IL-27 was also found to regulate IL-10 and IL-17 production by CD4^+^ cells [79]. Thus, IL-27 signalling appears to be important for the generation of IL-10 during experimental leishmaniasis, and one way this cytokine regulates host immune responses appears to involve regulating expression of PD-1 and its ligands.

Although IL-10-related pathways can explain important aspects of parasite persistence that promote the development of VL, other factors include changes to macrophage cell-signalling pathways. This area has been extensively reviewed [80]–[82] and is not covered in detail here except to highlight several major findings. *Leishmania* parasites can activate the macrophage protein tyrosine phosphotase SHP-1, which in turn inhibits the activation of Janus kinase 2 (JAK2) and extracellular signal-regulated kinases 1 and 2 (Erk1/2) signalling pathways [83]–[85]. SHP-1 also interferes with macrophage toll-like receptor (TLR) signalling by directly inactivating interleukin-1 receptor-associated kinase 1(IRAK-1) [86], [87]. Other important parasite-mediated changes include blocking protein kinase C activity [88]–[90], inhibition of NFκB and activator protein 1 (AP-1) transcriptional roles [91], [92], and suppression of JAK/STAT (signal transducer and activator of transcription) signalling pathways [83], [93]. Many of these manipulations are mediated by parasite cell surface molecules, such as lipophosphoglycan (LPG) and gp63 [94], [95], resulting in reduced inflammatory cytokine, RNI, and ROI generation (Figure 1), which enables parasite survival and growth within macrophages (Figure 2).

Relatively few studies have been conducted to investigate the effect of *L. donovani* infection on the BM in experimental VL. However, work by Cotterell et al. showed that in BALB/c mice, *L. donovani* affects the regulation of haematopoiesis [55]. Stromal macrophages in the BM were found to be targeted by *L. donovani*, and following exposure to granulocyte macrophage colony-stimulating factor (GM-CSF) and TNF, stromal macrophages were able to support increased level of myelopoiesis [55]. Related changes reported in VL-patient BM include an increase in plasma cell numbers, erythroid hyperplasia, and moderate-to-severe megaloblostosis [96].

Although studies in the spleen and BM of *L. donovani–*infected mice have provided a better understanding of the immune mechanisms associated with progressive and chronic infectious diseases, studies on disease models have limitations, and ultimately discoveries need to be validated in humans if they are going to be used to improve disease treatments or design better vaccines.

Recently, a model of natural VL has been established, whereby hamsters were infected with parasites transmitted by sand flies [97]. Although infection was delayed in comparison to intracardial injection of high parasite numbers, this model may prove useful in characterising the initiation and establishment of VL, particularly in defining the roles of various resident and recruited skin cell populations. Thus, this new model may shed new light on early immune events, while the mouse model of VL caused by high-dose intravenous injection of parasites might be more suitable for studying immune responses during established and/or chronic infection.

### Human VL

#### Disease susceptibility

As mentioned previously, the majority of the people infected with *L. donovani* never develop VL [4]–[6], [98]. The factors that influence susceptibility to VL are not fully understood. However, several genetic factors have been identified that are associated with susceptibility to VL. These include a major susceptibility locus on chromosome 22q12 [99] and polymorphisms in the *NRAMP1/Slc11ia* gene [100], [101]. However, these latter factors appear to have no role in VL affecting the Indian population [102]. Polymorphisms in the CXCR2 gene, which encodes receptors for IL-8 and other CXC chemokines, appear to play a role in determining VL outcome in Indian patients [103]. Polymorphisms in the IL-2Rβ gene, which is involved in T cell activation, are also implicated in determining VL susceptibility [104]. However, not only do polymorphisms in the human-leukocyte-antigen (HLA) genes play roles in susceptibility to experimental VL [105]–[107], but a recent study has also identified single nucleotide polymorphisms in this gene region that are strongly associated with both resistance and susceptibility to VL in Indian and Brazilian populations [108]. Some caution must be applied to these types of analysis because of reports of significant founder effects caused by rapid and recent migration of populations into VL-endemic regions [109]. More recently, the production of IL-17 and IL-22 by peripheral blood mononuclear cells (PBMCs) in response to heat-killed *L. donovani* was found to identify people protected from VL in the Sudan (see also below) [110]. Nutritional status can also influence disease susceptibility, with malnutrition being a major risk factor for VL, especially in rural settings [111]. Malnutrition negatively impacts on both cell-mediated and innate immunity [112], [113]. Furthermore, helminth infections are very common in these rural areas, which may favour *Leishmania* parasite replication [114], [115]. Other epidemiological factors, such as living in proximity to a previous VL patient, are also risk factors for developing VL [116]. More extensive discussions on epidemiological risk factors have been reviewed elsewhere [117]–[120].

#### The disease spectrum

Unlike experimental VL, in which there is a well-defined organ-specific course of infection, human VL manifests as a more heterogeneous form of disease with different levels of chronic infection observed in the spleen, liver, and BM [37]. Following the course of the infection in VL patients requires invasive techniques such as spleen and BM aspiration, which are uncomfortable, potentially dangerous, and time consuming. These techniques are still used for diagnostics purposes, and spare tissue provides rare opportunities to study disease manifestation in human target organs and to better understand human disease.

Most human *Leishmania* infections are subclinical or asymptomatic, and this can be attributed to the development of effective antiparasitic, cell-mediated immune responses [116], [121]. Only a small proportion of infected individuals develop disease, and VL patients that recover from infection are usually resistant to reinfection [116], [122]. Depressed cell-mediated immunity is a characteristic of human VL and is observed by negative leishmanin skin test and the failure of PBMCs to proliferate and produce IFNγ in response to *Leishmania* antigen [123]. In contrast, PBMCs taken from patients cured of VL are able to proliferate and produce IFNγ and TNF [123], suggesting that T cell responses in VL patients are refractory to antigenic stimulation [31]. However, several studies have shown that whole blood cells taken from active VL patients and stimulated with parasite antigen were able to produce elevated IFNγ at similar levels as those observed in cured VL patients, indicating that antigen-specific T cells were not refractory to stimulation but rather that other immunosuppressive factors might contribute to unfavourable clinical outcomes [123]–[125]. They also showed that significant amounts of IL-10 were produced by whole blood cells from VL patients in response to stimulation with parasite antigens in whole blood assays [123], [124].

#### Immune regulation

VL initially was thought to be associated with a dominant Th2-type immune response seen as elevated levels of IL-4 and/or IL-13 [126], [127]. Typically, VL is associated with increased production of multiple proinflammatory cytokines and chemokines. VL patients have elevated plasma protein levels of IL-1, IL-6, IL-8, IL-12, IL-15, IFNγ inducible protein-10 (IP-10), monokine induced by IFNγ (MIG), IFNγ, and TNF [126], [128]. Elevated levels of IFNγ mRNA have been found in the spleen and bone marrow during the acute phase of infection [126]. These observations suggest that unfavourable clinical outcomes are not related to Th2 skewing per se but that other mechanisms contribute to VL pathogenesis.

Studies on clinical samples have shown that elevated levels of IL-10 correlate with increased incidence of several human chronic infectious diseases, such as HIV, tuberculosis (TB), and malaria [129]–[132]. As mentioned earlier, IL-10 is an important regulatory cytokine that suppresses potentially damaging inflammatory immune responses [133]. However, these immunosuppressive properties of IL-10 can also target antigen presentation pathways in macrophages and DCs, thereby affecting T cell activation and cytokine production during chronic infection, potentially promoting parasite persistence [133]. VL patients have elevated levels of IL-10 in serum, and IL-10 mRNA accumulation was increased, relative to controls, in BM and spleen tissue [123]. IL-10 blockade in ex vivo cell assays using spleen tissue from VL patients showed increased IFNγ and TNF production associated with significantly reduced parasite growth [15], indicating that IL-10 is a major suppressor of leishmanicidal immune mechanisms in human VL patients (Figure 2). Other IL-10 neutralizing studies also showed enhanced IFNγ production by antigen-activated whole blood cells taken from VL patients [123]. A similar result was also found in studies on PBMCs from VL patients, in which increased IFNγ production, as well as enhanced T cell proliferation, was observed following IL-10 blockade [134]–[136]. The IL-10 in these human samples appeared to be produced predominantly by IFNγ-producing Tr1 cells [126]. However, another study recently showed that regulatory T (Treg) cells accumulated in the BM of VL patients and were a source of IL-10 that could suppress antiparasitic immunity [137].

Recent work by Ansari et al. showed elevated levels of circulating IL-27 and increased IL-27 mRNA accumulation in the spleen of VL patients, as well as enhanced expression of IL-21 mRNA [124]. IL-21 plays a role in amplifying IL-10 production by Tr1 cells induced by IL-27 [138]. The IL-27 and IL-21 in these samples appeared to be produced mainly by CD14^+^ (monocytes/macrophages) cells and CD3^+^ (T cells) cells, respectively [124]. Thus, these studies support the notion that IL-27 and IL-21 are key cytokines that promote the differentiation and expansion of antigen-specific IL-10-producing Tr1 cells during VL (Figure 2).

Human VL is also associated with a high level of plasma antibodies. Although sometimes useful in diagnosis, the role of antibodies in pathogenesis of VL is not clear. The high level of antibodies may drive the formation of immune complex, which can bind to the Fc receptors on macrophages, leading to the production of IL-10 by macrophages [139], and thus contribute to VL pathogenesis. Another cytokine, transforming growth factor beta (TGF-β), also has suppressive functions, and active VL is associated with increased plasma and mRNA levels of this cytokine [140]. The parasite-derived factor cathepsin-B, present in *L. donovani*, can activate TGF-β, which then has the potential to negatively impact on macrophage activity by lowering nitric oxide (NO) production [141], [142]. A better understanding of the precise mechanisms of TGF-β and IL-10 induction and activity during VL is required.

IL-17 has emerged as a potentially important cytokine in VL. A study in a Sudanese village during a VL outbreak over a 6-year period found that IL-17 and IL-22 production by PBMCs was tightly and independently associated with resistance to VL [110]. Thus, IL-17 and IL-22 may play complimentary roles to Th1 cytokines in controlling parasite growth and preventing the development of VL (Figure 1). The cellular mechanisms of parasite control induced by these cytokines remain unknown. Furthermore, the factors involved in blocking the production of these cytokines during active VL have not been fully elucidated, although IL-27 has been suggested to be involved in blocking Th17 expansion during infection [98]. Dissection of these processes should provide new insights into host control of parasite growth and resistance to VL.

The role of CD4^+^ T cells and Treg cells in human VL has been widely studied, but data on the role of CD8^+^ T cells are scarce. CD8^+^ T cells, like CD4^+^ cells, have immune regulatory capacity and can also directly kill the parasite-infected macrophages through cytolytic enzymes granzyme, granulysin, and perforin [143]–[145]. IL-10-producing CD8^+^ cells have been reported in human post-kala-azar dermal leishmaniasis (PKDL) and *L. guanyensis* infection [146], [147], while a recent study has shown that CD8^+^ T cells have an anergic or exhausted phenotype, as indicated by high expression of CTLA-4, PD-1, and IL-10, which may affect the protective capacity of these cells during clinical VL [148]. A better understanding of the role of CD8^+^ T cells in VL may help to harness the antiparasitic potential of these cells through vaccination or immune therapy.

#### PKDL

Post-kala-azar dermal leishmaniasis is a complication of VL characterised by a nodular, macular, or maculopapular rash on individuals who have recovered from VL. PKDL appears in individuals after apparently successful VL treatment and is possibly caused by suppression of immunity in the skin to persisting parasites [149], [150]. PDKL is mainly observed in the Indian subcontinent and East Africa, where an estimated 10%–20% of cases in India and 50%–60% of cases in the Sudan progress to PKDL after VL treatment [151]. Indian PKDL appears 2 to 7 years or even decades after the VL treatment, while in the Sudan it appears earlier (6 to 7 months after treatment) [151]. In some cases, there may be no previous history of leishmaniasis [152], [153]. PKDL cases are of epidemiological importance because these patients can serve as parasite reservoirs [151].

Immunological features of PKDL differ from VL in several ways [154]. In VL, a suppressed CMI response is observed, which is restored on successful treatment, and most cured individuals are resistant to reinfection [155]. PDKL, on the other hand, arises in a proportion of cured VL patients due to the suppression of immune response against *Leishmania* parasites present in the skin [156], [157]. PKDL cases studied in Sudan show an increase in CD3^+^ T cell infiltration within lesions containing *Leishmania* parasites or antigen, and IFNγ, IL-10, and IL-4 are the main cytokines produced in the inflamed lesions [158]. In another Sudanese study, Gasim et al. showed that PKDL could be predicted by assessment of IL-10, as higher levels of IL-10 were observed in plasma and keratinocytes of patients who developed PKDL compared to patients who did not [159]. A subsequent study by the same group also reported a positive association between the onset of PKDL and an increase in circulating parasite-specific PBMC, made evident by the stronger parasite-specific T cell responses [160]. In India, increased CMI responses were also observed in patients at the onset of PKDL compared to during chronic PKDL [161]. However, another study failed to find detectable antigen-specific immune responses in Indian PKDL patients [162], while Ganguly et al. reported that CMI responses were present in Indian PKDL patients but were dominated by antigen-specific IL-10 production by CD8^+^ T cells [146]. Clearly, further studies are required to both identify predisposing immune factors associated with PKDL development as well as to better define dysfunctional immune pathways operating during this serious disease complication.

CD4^+^ CD25^+^ Foxp3^+^ Treg cells are a subpopulation of CD4^+^ T cells involved in immune homeostasis with the potential to produce IL-10 during inflammation [163]. Studies by Katara et al. showed that Treg cell markers and IL-10 were elevated in tissue samples from PKDL patients when compared to tissue taken from healthy controls [164]. Furthermore, Treg cells were found to aggregate in tissue lesions of patients with PKDL in which there was a positive association between parasite burden, certain Treg cell markers, and IL-10 levels [164]. In another recent study, elevated IL-17, IL-23, and RORγt mRNA accumulation was found in PKDL lesions when compared with tissue after drug treatment, and this was accompanied by increased IL-17 and IL-23 plasma levels [165]. Thus, although PKDL is accompanied by IL-10-mediated immune suppression in many cases, the picture is not always clear and may also involve other deregulated inflammatory responses. Again, this is an area requiring further investigation at the molecular and cellular level.

#### HIV coinfection

Recent studies have shown that secondary infections are common in VL patients, possibly due to the marked immune suppression observed in infected patients [8]. *L.* donovani and *L. infantum* coinfection with HIV has now been recognised as a significant clinical problem [166]. Compared to other coinfections, a higher mortality rate has been reported in AIDS patients coinfected with *L. donovani* or *L. infantum* [167]. HIV patients with VL have enhanced proinflammatory cytokine responses, associated with increased HIV viral load, which can accelerate the progression from asymptomatic HIV to AIDS [168], [169]. In addition, parasite multiplication promotes survival, proliferation, and elevated levels of cellular dinucleotide triphosphate (dNTP) in human monocytes, which can also accelerate HIV replication [170], [171]. Treatment of VL in HIV patients involves the use of standard drugs, but due to the enhanced immune suppression in HIV patients and the partial reliance on host immune mechanisms for drug efficacy [149], these treatment strategies are often inadequate, and in most cases, patients are unresponsive to drug treatment [149]. Furthermore, those patients who do respond to treatment often relapse, possibly due to the low CD4^+^ T cells numbers associated with HIV infection and parasites persisting after drug treatment [172]. Host T cell responses are abrogated in HIV infection, including skewing away from Th1 responses [173] that are required for effective leishmanicidal responses [143]. Wolday et al. showed that PBMCs from *Leishmania*/HIV-coinfected individuals produce low levels of IL-12 and IFNγ and higher levels of IL-4 and IL-10 following stimulation with parasite antigen [174]. IL-15 is involved in promoting and maintaining Th1 responses and was also decreased in plasma from patients with *Leishmania*/HIV coinfection [175]. Hence, these results suggest that HIV infection suppresses the antiparasitic Th1 immune response required for parasite clearance [172].

### Future prospects

In the absence of a licensed human vaccine, new and improved VL therapies are required to reduce drug toxicity in patients and combat parasite drug resistance. Successful treatment of VL is dependent on host immune responses, and manipulation of these responses, alone or in combination with a drug, may be useful in improving VL treatment. Immune modulation aimed at improving host immune responses may be one way to enhance *Leishmania-*specific immune response, in the presence or absence of conventional therapy, thereby allowing lower drug doses or shorter drug treatment periods, as well as reducing the risk of drug-resistant parasites emerging. IFNγ in combination with pentavalent antimonial has been used in the past for treating VL and diffused CL [176]. However, targeting regulatory molecules or other cytokines might result in better outcomes. Studies on regulatory immune molecules in mice with established experimental infection and in VL patients may identify suitable targets for such immune modulation. As discussed above, IL-10 is one such candidate identified by these approaches, since inhibiting or neutralizing IL-10 results in improved immune regulation and parasite killing both in mice and humans [15], [70], [71]. A number of other promising targets have also been identified [73], [177], and it is anticipated that many more soon will be. One of the major challenges will be identifying strategies for immune modulation that are affordable and suitable for implementation in the clinical setting where VL patients must be treated.

*Leishmania* vaccine research is another area which requires further work, particularly given that available epidemiological and historical data indicate that an effective vaccine is a realistic goal. A major challenge in developing such a vaccine is the necessity of inducing a strong and lasting CMI response. Most *Leishmania* vaccines that reach clinical trials have been unable to initiate strong T cell responses [31], possibly due to relatively poor adjuvants used in vaccine formulations. In addition, there is an urgent need for good surrogate markers of immunity so that vaccine candidates can be effectively evaluated in a timely manner. Nevertheless, parasite antigens for vaccines that show protective efficacy against *L. donovani* or *L. infantum* infection in experimental VL models have recently been evaluated for use in humans [178]. In addition, clinical trials have begun to test promising vaccine candidates [179]–[181]. This is now an active area of research, and with our increasing knowledge of protective immune mechanisms required to prevent the onset of VL, it is likely that suitable adjuvants can be developed for combining with vaccines to induce long-lasting protection against VL in the near future. Again, making these vaccines affordable and deliverable will be a major challenge.

**Box 1. Key Learning Points**

- The majority of people infected with *L. donovani* and *L. infantum* rapidly control parasite growth and do not develop visceral leishmaniasis.
- The host immune response to persisting parasites is a major cause of disease during visceral leishmaniasis.
- Visceral leishmaniasis encompasses a disease spectrum involving varying levels of tissue-specific immunity and immune-mediated tissue damage.
- Antiparasitic, cell-mediated immune responses can be activated in visceral leishmaniasis patients and enhanced following blockade of negative immune regulators.
- CD4^+^ T cell–derived IL-10 is a key immune regulator during visceral leishmaniasis and a potential clinical target.

**Box 2. Top Five Papers in the Field**

- Stern J, Oca M, Rubin B, Anderson S, Murray H (1988) Role of L3T4^+^ and LyT-2^+^ cells in experimental visceral leishmaniasis. J Immunol 140: 3971–3977.
- Nylén S, Maurya R, Eidsmo L, Manandhar K, Sundar S, et al. (2007) Splenic accumulation of IL-10 mRNA in T cells distinct from CD4+CD25+ (Foxp3) regulatory T cells in human visceral leishmaniasis. J Exp Med 204: 805–817.
- Dalton J, Maroof A, Owens B, Narang P, Johnson K, et al. (2010) Inhibition of receptor tyrosine kinases restores immunocompetence and improves immune-dependent chemotherapy against experimental leishmaniasis in mice. J Clin Invest 120: 1204–1216.
- Gautam S, Kumar R, Maurya R, Nylén S, Ansari N, et al. (2011) IL-10 neutralization promotes parasite clearance in splenic aspirate cells from patients with visceral leishmaniasis. J Infect Dis 204: 1134–1137.
- Fakiola M, Strange A, Cordell HJ, Miller EN, Pirinen M, et al. (2013) Common variants in the HLA-DRB1-HLA-DQA1 HLA class II region are associated with susceptibility to visceral leishmaniasis. Nat Genet 45: 208–213.
